# Vinorelbine With or Without Thiotepa for HER2‐Negative Metastatic Breast Cancer: A Propensity Score Analysis

**DOI:** 10.1002/cam4.71102

**Published:** 2025-07-28

**Authors:** Aurelia Robert, Paul Gougis, Elise Dumas, Rebecca Loison, Victoire De Castelbajac, Marc Espie, Sylvie Giacchetti, Caroline Cuvier, Lamia Hassani, Johanna Wassermann, Luca Campedel, Marianne Veyri, Aurore Vozy, Jean Philippe Spano, Luis Teixeira, Baptiste Abbar

**Affiliations:** ^1^ Department of Medical Oncology Sorbonne Université, Assistance Publique—Hôpitaux de Paris (AP‐HP), Institut Universitaire de Cancérologie, CLIP^2^ Galilée, Pitié‐Salpêtrière Hospital Paris France; ^2^ Sorbonne Université, Department of Pharmacology CIC Paris‐Est, INSERM, Pitié‐Salpêtrière Hospital Paris France; ^3^ Institut Curie, Residual Tumor & Response to Treatment Laboratory, RT2Lab INSERM U932 Immunity and Cancer Paris France; ^4^ Department of Senology Université Paris Cité, AP‐HP, Saint‐Louis Hospital Paris France; ^5^ SorbonneUniversité, AP‐HP, Pitié‐SalpêtrièreHospital, Department of Pharmacy Paris France; ^6^ Department ofMedical and Thoracic Oncology, CHU Gabriel Montpied Clermont‐Ferrand France; ^7^ Sorbonne Université, INSERM U1135, Centre d'Immunologie et des Maladies Infectieuses de Paris (CIMI‐Paris) Paris France

**Keywords:** breast cancer, propensity score, real‐world evidence, thiotepa, vinorelbine

## Abstract

**Background:**

Vinorelbine is commonly used to treat metastatic breast cancer (mBC), while thiotepa is known for its ability to cross the blood–brain barrier.

**Methods:**

Our retrospective study aimed to compare the efficacy and safety of vinorelbine with or without thiotepa in patients with HER2‐negative mBC. We used propensity score inverse probability of treatment weighting to ensure comparability between groups.

**Results:**

Vinorelbine‐thiotepa was not significantly associated with improved median progression‐free survival (PFS) (4.9 vs. 3.0 months, *p* = 0.138) or median overall survival (OS) (11.8 vs. 11.9 months, *p* = 0.961) compared to vinorelbine. However, in the central nervous system (CNS) metastasis subgroup, vinorelbine‐thiotepa was associated with a longer median PFS (4.9 vs. 2.1 months, *p* = 0.013) and CNS‐PFS (6.12 vs. 2.20 months, *p* = 0.007). The combination was also associated with a higher rate of grade ≥ 3 adverse events (54.3% vs. 37.9%, *p* = 0.021).

**Conclusion:**

While no overall benefit in PFS or OS was found, vinorelbine‐thiotepa may be associated with improved PFS in mBC patients with CNS metastasis.

## Graphical abstract

Comparative analysis of thiotepa plus vinorelbine versus vinorelbine alone in patients with HER2‐negative metastatic breast cancer, using a propensity score emulated target trial. While no progression‐free survival benefit was observed in the overall population, vinorelbine–thiotepa was associated with significantly improved median progression‐free survival in patients with central nervous system metastases.

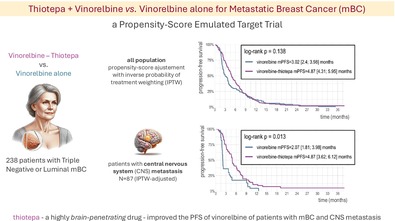

**Abbreviations**
CNS: central nervous system
IPTW: inverse probability of treatment weighting
mBC: metastatic breast cancer
mPFS: median progression‐free survival

## 1 Introduction

Breast cancer remains the most common and deadly cancer among women worldwide [1]. In metastatic breast cancer (mBC), survival rates are influenced by numerous factors such as metastasis location, with central nervous system (CNS) metastasis posing a particular challenge due to the blood–brain barrier, which limits the effectiveness of many drugs [2, 3].

Vinorelbine, a vinca‐alkaloid, is frequently used to treat mBC, showing an objective response rate of approximately 40% when used as monotherapy [4]. Thiotepa, an organophosphorus alkylating agent, is known for its ability to cross the blood–brain barrier due to its lipophilic structure and is used in treating various malignancies [5]. Thiotepa has been studied in combination regimens for nonmetastatic or locally advanced breast cancer, though these data are over two decades old and lack phase III validation [6, 7]. The combination of vinorelbine and thiotepa has also been evaluated in a Phase II trial for the treatment of mBC, which showed an objective response rate (ORR) of 28% and median progression‐free survival (mPFS) of 6 months in the second‐line setting [8]. A retrospective study involving 137 mBC patients showed an mPFS of 4.4 months and median overall survival (mOS) of 12.7 months [9]. However, no comparative study has assessed the efficacy and safety of this chemotherapy combination for mBC.

In this real‐world study, we aim to compare the outcomes of vinorelbine alone versus vinorelbine with thiotepa in HER2‐negative mBC patients, using propensity score inverse probability of treatment weighting (IPTW) analysis. The primary objective was to evaluate PFS, with secondary outcomes including OS and toxicity profile.

## 2 Materials and Methods

### 2.1 Study Design and Population

Data were retrospectively collected from patients with mBC who received vinorelbine‐thiotepa or vinorelbine monotherapy between April 2010 and September 2023 at Saint‐Louis Hospital or Pitié‐Salpêtrière Hospital, Paris, France. Eligible patients had histologically confirmed breast cancer and had received at least one prior chemotherapy line with anthracyclines or taxanes. Inclusion criteria also required patients to have received at least one full cycle of vinorelbine‐thiotepa or vinorelbine monotherapy and to be ≥ 18 years old. Exclusion criteria included treatment prior to 2010, HER‐2 positive mBC, concurrent antitumor therapies, follow‐up < 14 days, other concurrent malignancies, or nonmetastatic breast cancer.

### 2.2 Study Treatment

Vinorelbine monotherapy was given at 30 mg/m^2^ intravenously on days 1 and 8 every 3 weeks. In the combination group, vinorelbine (30 mg/m^2^) and thiotepa (12 mg/m^2^) were administered intravenously on days 1 and 8, every 3 weeks. Patients with CNS metastasis could receive additional intrathecal chemotherapy, local radiotherapy, or surgery. Vinorelbine‐based chemotherapy was generally considered in both centers for patients with HER2‐negative mBC previously treated with taxanes and/or anthracyclines. Treatment allocation followed institutional practices: Vinorelbine‐thiotepa was standard at Saint‐Louis Hospital except for frail patients, while vinorelbine alone was preferred at Pitié‐Salpêtrière, with the combination reserved for select cases with multiple CNS metastases.

### 2.3 Endpoints

The primary endpoint was PFS, defined as the time from vinorelbine initiation to disease progression (RECIST or non‐RECIST radiological progression) or death. Subgroup analyses included PFS in patients with CNS metastasis, hormone receptor‐positive (HR+), and triple‐negative breast cancer (TNBC). As a secondary endpoint, we also analyzed CNS‐PFS in the subgroup of patients with CNS metastases. CNS‐PFS was defined as the time to CNS progression or death, with patients censored at the date of extra‐CNS progression without CNS progression or at last follow‐up if no event had occurred. Other secondary endpoints were OS and safety. Adverse events were retrospectively collected and graded using the National Cancer Institute Common Terminology Criteria for Adverse Events (CTCAE), version 5.0.

### 2.4 Statistical Analysis

We used IPTW to adjust for confounding. First, a directed acyclic graph was drawn based on expert knowledge to identify confounders: age, number of prior metastatic lines, HR status, brain metastasis, and carcinomatous meningitis (Figure S1). Next, a logistic regression model was fitted to estimate the propensity score, representing the probability of receiving vinorelbine‐thiotepa. Patients were then weighted based on the inverse of their propensity score. The average treatment effect (ATE) was calculated as the difference in mPFS and mOS in the weighted population and visualized with weighted Kaplan–Meier curves and log‐rank tests. Subgroup analyses were performed by CNS status and HR status (HR+ or TNBC). Complications were compared using Fisher or Wilcoxon tests. Statistical analyses were performed with R 4.3.1.

### 2.5 Ethics Statement

All living patients provided oral consent for data collection after receiving written information. Clinical records were retrospectively retrieved in a de‐identified format. This study was approved by the Ethical Review Board of the French College of Gynecologists and Obstetricians (2022‐GYN‐0304). We followed the STROBE guidelines to ensure a transparent and thorough presentation of our methodology and results [10].

## 3 Results

### 3.1 Patient Characteristics

A total of 610 patients with breast cancer and vinorelbine‐based treatment were identified through our database. Among them, 372 patients were excluded, 14 did not meet eligibility criteria, and 358 presented exclusion criteria (mainly for receiving other concurrent antitumor treatments). A total of 238 patients were included, with 87 treated with vinorelbine and 151 treated with vinorelbine‐thiotepa (Figure S2: flowchart).

The median age was 53.3 years (range 31–86), with 99.2% of patients being women. The most common histological type was invasive ductal carcinoma (86.1%). Patients had a median of 2 prior systemic regimens (range 0–11). Of the 238 patients, 176 (73.9%) were HR+ and 62 (26.1%) were TNBC. Sixty‐seven (28.2%) had brain metastasis, and 31 (13%) had leptomeningeal carcinomatosis.

Compared to the vinorelbine group, patients in the vinorelbine‐thiotepa group were younger (median age 56.7 vs. 61 years), primarily treated at Saint‐Louis Hospital (98.7% vs. 14.9%), had a higher proportion of invasive ductal carcinoma (90.7% vs. 78.2%) and TNBC (31.8% vs. 16.1%), and more frequently underwent prior breast surgeries (96.7% vs. 80.5%) and radiotherapy (88.1% vs. 75.9%). They also received fewer lines of systemic treatment (59.6% vs. 40.2% for 0–2 prior lines) and had a higher incidence of brain metastasis (33.1% vs. 19.5%, *p* = 0.036). The patients' characteristics are detailed in Table 1. Propensity score distribution by treatment group is available in Figure S3 and quality check for IPTW is available in Figure S4.

**TABLE 1:** Patient's characteristics.

Features	Level	Overall	Vinorelbine	Vinorelbine‐thiotepa	*p*
	*n*	238	87 (36.6)	151 (63.4)	
Center	Pitié‐Salpêtrière (%)	76 (31.9)	74 (85.1)	2 (1.3)	**< 0.001**
	Saint Louis (%)	162 (68.1)	13 (14.9)	149 (98.7)	
Sex	F (%)	236 (99.2)	86 (98.9)	150 (99.3)	1.000
	M (%)	2 (0.8)	1 (1.1)	1 (0.7)	
Age (years)	Median (IQR)	58.3 (12.7)	61.0 (12.9)	56.7 (12.3)	**0.013**
Year of treatment initiation	2010–2014 (%)	78 (32.8)	30 (34.5)	48 (31.8)	0.611
	2015–2019 (%)	105 (44.1)	40 (46.0)	65 (43.0)	
	2020–2023 (%)	55 (23.1)	17 (19.5)	38 (25.2)	
Histology	IDC	205 (86.1)	68 (78.2)	137 (90.7)	**< 0.001**
	ILC	25 (10.5)	18 (20.7)	7 (4.6)	
	Other	8 (3.4)	1 (1.1)	7 (4.6)	
Hormone receptor expression	Negative	62 (26.1)	14 (16.1)	48 (31.8)	**0.012**
	Positive	176 (73.9)	73 (83.9)	103 (68.2)	
Synchronous or metachronous metastasis	Metachronous	183 (76.9)	66 (75.9)	117 (77.5)	0.900
	Synchronous	55 (23.1)	21 (24.1)	34 (22.5)	
Perioperative chemotherapy	No	67 (28.2)	29 (33.3)	38 (25.2)	0.230
	Yes	171 (71.8)	58 (66.7)	113 (74.8)	
Adjuvant endocrine therapy	No	101 (45.5)	35 (43.2)	66 (46.8)	0.705
	Yes	121 (54.5)	46 (56.8)	75 (53.2)	
Breast cancer surgery	No	22 (9.2)	17 (19.5)	5 (3.3)	**< 0.001**
	Yes	216 (90.8)	70 (80.5)	146 (96.7)	
Breast cancer radiotherapy	No	39 (16.4)	21 (24.1)	18 (11.9)	**0.023**
	Yes	199 (83.6)	66 (75.9)	133 (88.1)	
Endocrine therapy for mBC	No	79 (33.2)	24 (27.6)	55 (36.4)	0.211
	Yes	159 (66.8)	63 (72.4)	96 (63.6)	
CDK4/6 inhibitors for mBC	No	169 (71.0)	62 (71.3)	107 (70.9)	1.000
	Yes	69 (29.0)	25 (28.7)	44 (29.1)	
Everolimus for mBC	No	177 (74.4)	66 (75.9)	111 (73.5)	0.806
	Yes	61 (25.6)	21 (24.1)	40 (26.5)	
Number of previous metastatic lines	0–2	125 (52.5)	35 (40.2)	90 (59.6)	**0.014**
	3–5	83 (34.9)	37 (42.5)	46 (30.5)	
	6+	30 (12.6)	15 (17.2)	15 (9.9)	
Extra‐CNS metastatic sites	Bone only	38 (16.0)	20 (23.3)	18 (11.9)	0.073
	Bone and visceral	139 (58.6)	46 (53.5)	93 (61.6)	
	Visceral only	60 (25.3)	20 (23.3)	40 (26.5)	
Brain metastasis	No	171 (71.8)	70 (80.5)	101 (66.9)	**0.036**
	Yes	67 (28.2)	17 (19.5)	50 (33.1)	
Leptomeningeal carcinomatosis	No	207 (87.0)	74 (85.1)	133 (88.1)	0.640
	Yes	31 (13.0)	13 (14.9)	18 (11.9)	
Prior CNS treatment	Radiotherapy	28 (11.8)	4 (4.6)	24 (15.9)	**0.009**
	Surgery	7 (2.9)	1 (1.1)	6 (4.0)	0.4
	Intrathecal chemotherapy	5 (2.1)	0 (0)	5 (3.3)	0.2

*Note:* *p*‐value calculated from Fischer tests. Bold values indicate *p*‐values < 0.05.

Abbreviations: CDK, cyclin dependent kinase; CNS, central nervous system; IDC, invasive ductal carcinoma; ILC, invasive lobular carcinoma; MBC, metastatic breast cancer.

### 3.2 Efficacy

**FIGURE 1:**
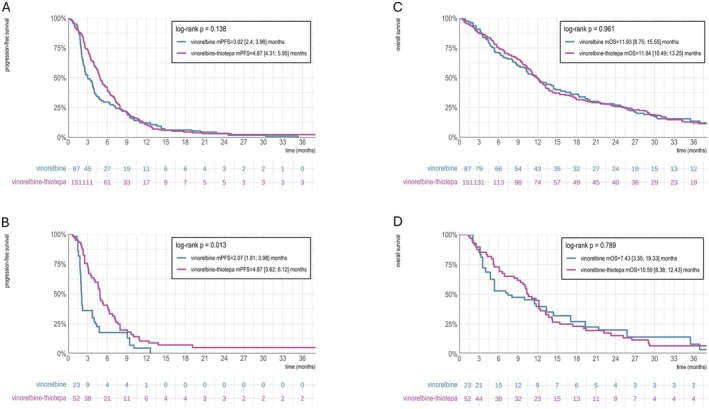
Progression‐Free Survival and Overall Survival in the IPTW‐adjusted overall population and CNS‐metastasis population. Kaplan–Meier progression‐free survival (PFS) curves for the (A) IPTW‐adjusted overall population and the (B) IPTW‐adjusted CNS‐metastasis population. Kaplan–Meier overall survival (OS) curves for the (C) IPTW‐adjusted overall population and the (D) IPTW‐adjusted CNS‐metastasis population. CNS, Central nervous system; IPTW, Inverse probability of treatment weighting; OS, Overall survival; PFS, Progression‐free survival.

In the overall population, mPFS was 4.87 months in the vinorelbine‐thiotepa group and 3.02 months in the vinorelbine group (*p* = 0.138) (Figure 1A). In the CNS population, mPFS was longer in the vinorelbine‐thiotepa group (4.87 vs. 2.07 months, *p* = 0.013) (Figure 1B). In the TNBC and HR+ subgroups, no significant PFS differences were observed (TNBC: 3.95 vs. 2.30 months, *p* = 0.595; HR+: 5.23 vs. 3.45 months, *p* = 0.2) (Figure S5A,B). Regarding OS, the vinorelbine‐thiotepa group had an mOS of 11.84 months, while the vinorelbine group had 11.93 months (*p* = 0.961) (Figure 1C). In the CNS population, mOS was 10.59 months for vinorelbine‐thiotepa and 7.43 months for vinorelbine (*p* = 0.789) (Figure 1D). Regarding CNS‐PFS in the CNS population, the median CNS‐PFS was longer in the vinorelbine‐thiotepa group compared to the vinorelbine group (6.12 vs. 2.20 months, *p* = 0.007) (Figure S6). For concomitant CNS treatments, encephalic irradiation was given to 8.0% of the vinorelbine group and 14.6% of the vinorelbine‐thiotepa group (*p* = 0.15), brain surgery to 0% and 1.3% (*p* = 0.53), and intrathecal chemotherapy to 8.0% and 5.3% (*p* = 0.41) (Table S1).

### 3.3 Safety

Grade ≥ 3 treatment‐related adverse events occurred in 33/87 (37.9%) patients in the vinorelbine group and 82/151 (54.3%) in the vinorelbine‐thiotepa group (*p* = 0.021). Hematological adverse events were more common in the vinorelbine‐thiotepa group (39.1% vs. 20.7%, *p* = 0.003). Dose reductions due to adverse events were required for 19.5% of vinorelbine and 37.7% of vinorelbine‐thiotepa patients (*p* = 0.004). Treatment discontinuation occurred in 8% and 10.6% of patients, respectively (*p* = 0.5). Three patients died from treatment‐related septic shock (1.1% in vinorelbine, 1.3% in vinorelbine‐thiotepa, *p* = 1.000). Safety details are in Table S2.

## 4 Discussion

This real‐world data study aimed to assess the efficacy and safety profile of adding thiotepa to vinorelbine for the management of patients with HER2‐negative mBC. In this cohort, we found a tendency toward an increased PFS with thiotepa added to vinorelbine that did not meet significance in the overall population. There was no improvement in OS. However, our results suggest that combining vinorelbine with thiotepa may increase PFS in patients with CNS metastasis.

Empirical chemotherapies such as anthracyclines and taxanes remain essential in managing both early and mBC. Guidelines typically recommend sequential cytotoxic monotherapy for mBC, which often provides satisfactory disease control [11, 12]. Polychemotherapy aims to enhance the ORR and disease control, particularly in aggressive cancers.

Vinorelbine is widely used for mBC, and various combinations have proven effective [4]. The vinorelbine‐thiotepa combination was explored in a phase II trial without a comparative group [8]. More recently, our group published an observational retrospective study with 137 patients with mBC treated with this combination [9]. Our initial study's interpretation was constrained by the absence of a comparative group receiving single‐agent vinorelbine. To address this limitation, we expanded our retrospective cohort from the Saint‐Louis Hospital and added the Pitié‐Salpêtrière Hospital to introduce a control group receiving vinorelbine monotherapy.

Although adding thiotepa increased toxicity, particularly hematological adverse events, it did not improve PFS or OS in the overall population. Subgroup analysis showed no benefit in HR+ or TNBC patients. In the CNS metastasis subgroup, thiotepa was associated with extended PFS, likely due to its ability to cross the blood–brain barrier, a point further supported by the observed prolongation of CNS PFS with the vinorelbine‐thiotepa combination. Importantly, none of the patients in this subgroup had received prior systemic agents known to penetrate the CNS, such as tucatinib or trastuzumab deruxtecan, limiting potential confounding of thiotepa's observed effect. Although thiotepa is not currently recommended in major international guidelines such as the National Comprehensive Cancer Network (NCCN) for the treatment of mBC, its use in our cohort reflects a longstanding institutional practice initiated after encouraging results from an earlier phase II study [8]. While this limits the applicability of the regimen in standardized care pathways, our study contributes to the current field by providing the first comparative analysis of this combination versus standard vinorelbine monotherapy, particularly in the challenging setting of CNS metastases. These findings may inform future research on CNS‐penetrant regimens and highlight the need for prospective trials assessing less commonly used agents that may have indications in specific subgroups.

The study has limitations due to its retrospective nature, missing data, and variability in radiological evaluations. An important limitation is the absence of best radiological response evaluation according to Response Evaluation Criteria in Solid Tumors (RECIST), which could not be performed due to variability in radiological assessments and the lack of available imaging data. Moreover, HER2 expression status (low vs. absent) was unavailable for a substantial proportion of patients, although none received anti‐HER2 targeted therapy at any point. Significant heterogeneity was observed in prior chemotherapy regimens and tumor types. Additionally, nearly all patients receiving vinorelbine‐thiotepa were treated at the Saint‐Louis Hospital, which we could not include in the propensity score model due to this high center‐related heterogeneity. The concurrent CNS treatments in the CNS metastasis subgroup also introduce potential confounding bias.

This study represents the first reported comparison between vinorelbine monotherapy and vinorelbine‐thiotepa for the treatment of patients with mBC. Our findings indicate potential promise for vinorelbine‐thiotepa in patients with CNS metastasis. However, the notable toxicity linked to this combination highlights the imperative of careful patient selection. While recognizing the inherent limitations of our study, we emphasize the importance of future prospective randomized clinical trials to thoroughly evaluate the viability of this treatment strategy.

## Author Contributions

**Aurelia Robert:** investigation, formal analysis, and writing – original draft. **Paul Gougis:** conceptualization, methodology, software, formal analysis, data curation, and writing – review and editing. **Elise Dumas:** methodology, software, formal analysis, data curation, writing – review and editing. **Rebecca Loison:** investigation and writing – review and editing. **Victoire De Castelbajac:** project administration, resources, and writing – review and editing. **Marc Espie:** resources and writing – review and editing. **Sylvie Giacchetti:** resources and writing – review and editing. **Caroline Cuvier:** resources and writing – review and editing. **Lamia Hassani:** resources and writing – review and editing. **Johanna Wassermann:** resources and writing – review and editing. **Luca Campedel:** resources and writing – review and editing. **Marianne Veyri:** resources and writing – review and editing. **Aurore Vozy:** resources and writing – review and editing. **Jean Philippe Spano:** supervision, resources, and writing – review and editing. **Luis Teixeira:** supervision, resources, and writing – review and editing. **Baptiste Abbar:** project administration, conceptualization, supervision, methodology, and writing – review and editing.

## Disclosure

P.G. reports consulting fees for BMS, an academic grant from Sanofi, and travel accommodation by Eisai. M.E. reports consultant from Roche. S.G. reports consultant or advisory role fees or travel accommodations from Novartis, EISAI, MSD, and Viatris. J.W. reports consultant or advisory from EISAI, Menarini, Lilly, Exact Sciences, and Novartis. L.C. reports consultant or advisory role fees or travel accommodations from AAA, Amgen, Astellas, Bayer, BMS, Eisai, Ipsen, Janssen, Merck, MSD, and Pfizer. M.V. reports honoraria from Gilead. A.V. reports consultant from Merck Serrano, MSD and travel accommodations from Amgen. J.P.S. reports consultant or advisory role fees from Roche, MSD, BMS, Lilly, AstraZeneca, Daiichi‐Sankyo, Mylan, Novartis, Pfizer, PFO, LeoPharma, and Gilead and Grant for MSD Avenir. L.T. reports consultant or advisory role fees or travel accommodations or research grants from AZD, Daiichi, Gilead, MSD, Novartis, Pfizer, Roche, Pfizer, and Mylan. B.A. reports research grant from MSD avenir, and consulting fees or honoraria from Novartis, AstraZeneca, BMS, MSD, Astellas, and Sanofi. A.R., E.D., R.L., V.D.C., C.C., and L.H. declare no conflicts of interest.

## Ethics Statement

All alive patients received written information and provided their oral consent for data collection. Patients' clinical records were retrospectively retrieved from electronic files using a de‐identified format. This study received approval from the Ethical Review Board of the French College of Gynecologists and Obstetricians (2022‐GYN‐0304). Following the STROBE (Strengthening the Reporting of Observational Studies in Epidemiology) guidelines, we have organized the reporting of our study to ensure a thorough and transparent presentation of our research methodology and results.

## Conflicts of Interest

The authors declare no conflicts of interest.

## Supporting information


**Appendix S1:** cam471102‐sup‐0001‐AppendixS1.docx.

## Data Availability

The data that support the findings of this study are available from the corresponding author upon reasonable request.
