# Supplementary material for: Vinorelbine With or Without Thiotepa for HER2‐Negative Metastatic Breast Cancer: A Propensity Score Analysis
Source: Cancer Med. 2025 Jul 28;14(15):e71102. doi: 10.1002/cam4.71102 (PMC12301934; doi:10.1002/cam4.71102)
Supplement: Supplementary file 1 — Appendix S1: cam471102‐sup‐0001‐AppendixS1.docx. [file CAM4-14-e71102-s001.docx]

**RESEARCH ARTICLE**

**Vinorelbine with or without Thiotepa for HER2-negative Metastatic Breast Cancer: A Propensity Score Analysis**

**Supplementary Tables and Figures**

[**Supplementary Table 1: concomitant CNS treatment** 2](#_Toc200105320)

[**Supplementary Table 2: safety profile: CTCAE grade≥3 adverse events:** 3](#_Toc200105321)

[**Supplementary Figure 1: directed acyclic graph.** 4](#_Toc200105322)

[**Supplementary Figure 2: flow chart** 5](#_Toc200105323)

[**Supplementary Figure 3: propensity score distribution by treatment group.** 6](#_Toc200105324)

[**Supplementary Figure 4: quality check for inverse probability of treatment weighting** 7](#_Toc200105325)

[**Supplementary Figure 5: progression-free survival in the IPTW-adjusted hormone receptor-positive and the triple negative populations:** 8](#_Toc200105326)

[**Supplementary Figure 6: central nervous system progression-free survival (CNS-PFS) in the IPTW-adjusted CNS-metastasis population:** 9](#_Toc200105327)

# **Supplementary Table 1: concomitant CNS treatment**

| Concomitant CNS treatment | overall | vinorelbine | vinorelbine-thiotepa | p-value |
| --- | --- | --- | --- | --- |
| n (%) | 238 | 87 (36.6) | 151 (63.4) |  |
| radiotherapy | 29 (12.2) | 7 (8.0) | 22 (14.6) | 0.15 |
| intrathecal chemotherapy | 15 (6.3) | 7 (8.0) | 8 (5.3) | 0.41 |
| surgery | 2 (0.8) | 0 (0) | 2 (1.3) | 0.53 |

p-value calculated from Fisher test

CNS: central nervous system

# **Supplementary Table 2: safety profile: CTCAE grade≥3 adverse events:**

| CTCAE v 5.0 grade≥3 | overall | vinorelbine | vinorelbine-thiotepa | p-value |
| --- | --- | --- | --- | --- |
|  | 238 | 87 (36.6) | 151 (63.4) |  |
| any adverse-event grade≥3 | 115 (48.3) | 33 (37.9) | 82 (54.3) | **0.021** |
| hematotoxicity | 77 (32.4) | 18 (20.7) | 59 (39.1) | **0.003** |
| digestive toxicity | 16 (6.7) | 4 (4.6) | 12 (7.9) | 0.42 |
| peripheral neuropathy | 13 (5.5) | 4 (4.6%) | 9 (6) | 0.77 |
| Others^†^ | 16 (6.7) | 7 (8) | 9 (6) | 0.59 |
| dose reduction | 74 (31) | 17 (19.5) | 57 (37.7) | **0.004** |
| cause of dose reduction:  hematotoxicity  peripheral neuropathy  digestive toxicity  PS decline  ASAT or ALAT increased  sepsis  unknown | 39 (16.4)  9 (3.8)  5 (2.1)  2 (0.8)  1 (0.4)  1 (0.4)  17 (7.1) | 8 (9.2)  2 (2.3)  1 (1.1)  1 (1.1)  -  -  5 (5.7) | 31 (20.5)  7 (4.6)  4 (2.6)  1 (0.7)  1 (0.7)  1 (0.7)  12 (8) |  |
| discontinuation due to toxicity | 23 (9.7) | 7 (8) | 16 (10.6) | 0.5 |
| toxic death^‡^ | 3 (1.3) | 1 (1.1) | 2 (1.3) | 1.000 |

p-value calculated from Fisher test

CTCAE v 5.0: Common Terminology Criteria for Adverse Events version 5.0; PS: Performance status; ASAT : Aspartate-aminotransferase; ALAT : alanine-aminotransferase

† Including: Aspartate-aminotransferase or alanine-aminotransferase increased, sepsis and asthenia.

‡ Toxic deaths: 3 sepsis shock.

# **Supplementary Figure 1: directed acyclic graph.**


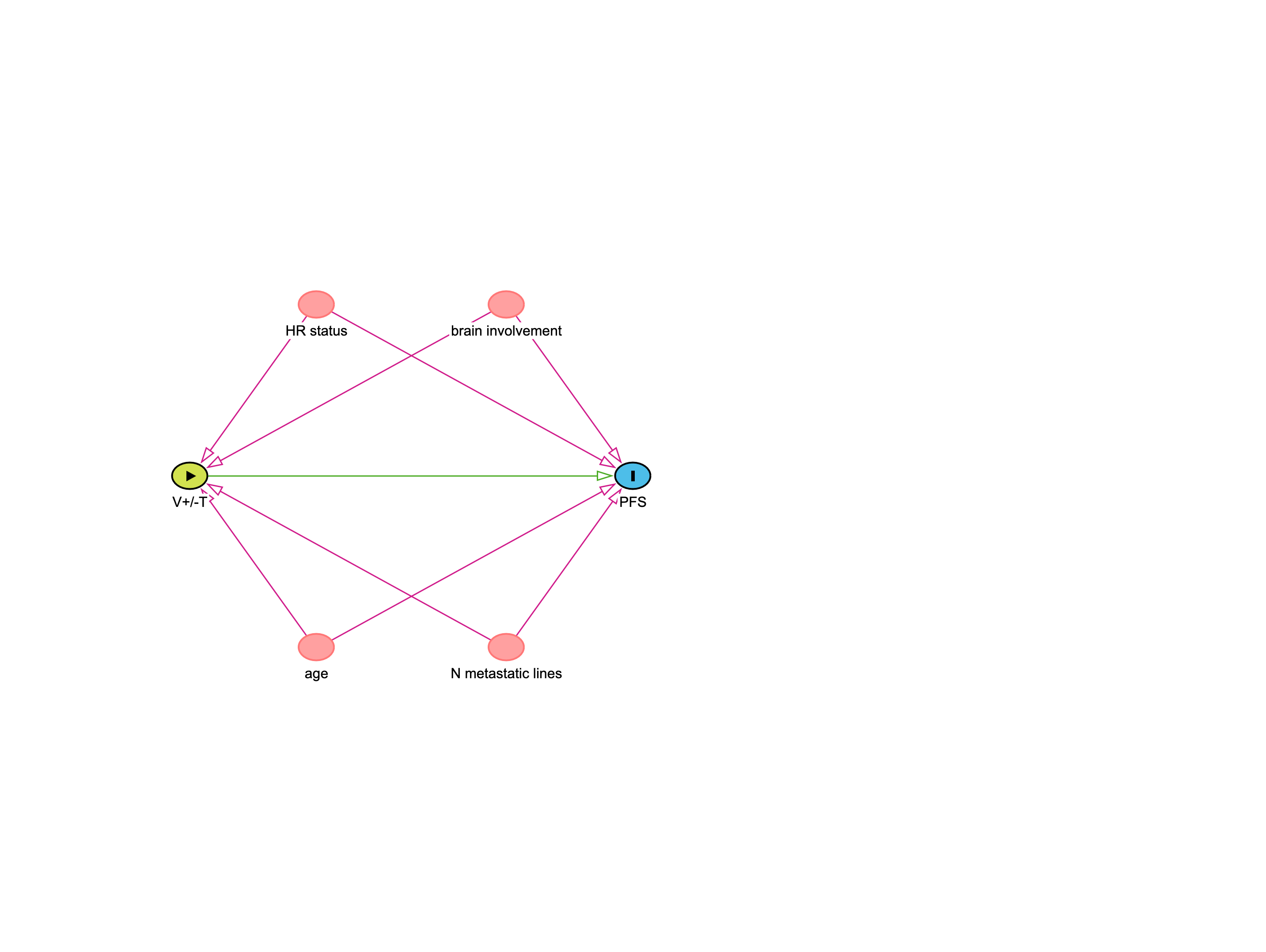


Directed acyclic graph used to identify the covariates included in the propensity score model for adjustment by inverse probability of treatment weighting.

Abbreviations: V+/-T: vinorelbine with or without thiotepa; PFS: progression-free survival ; HR: hormone receptor

# **Supplementary Figure 2: flow chart**


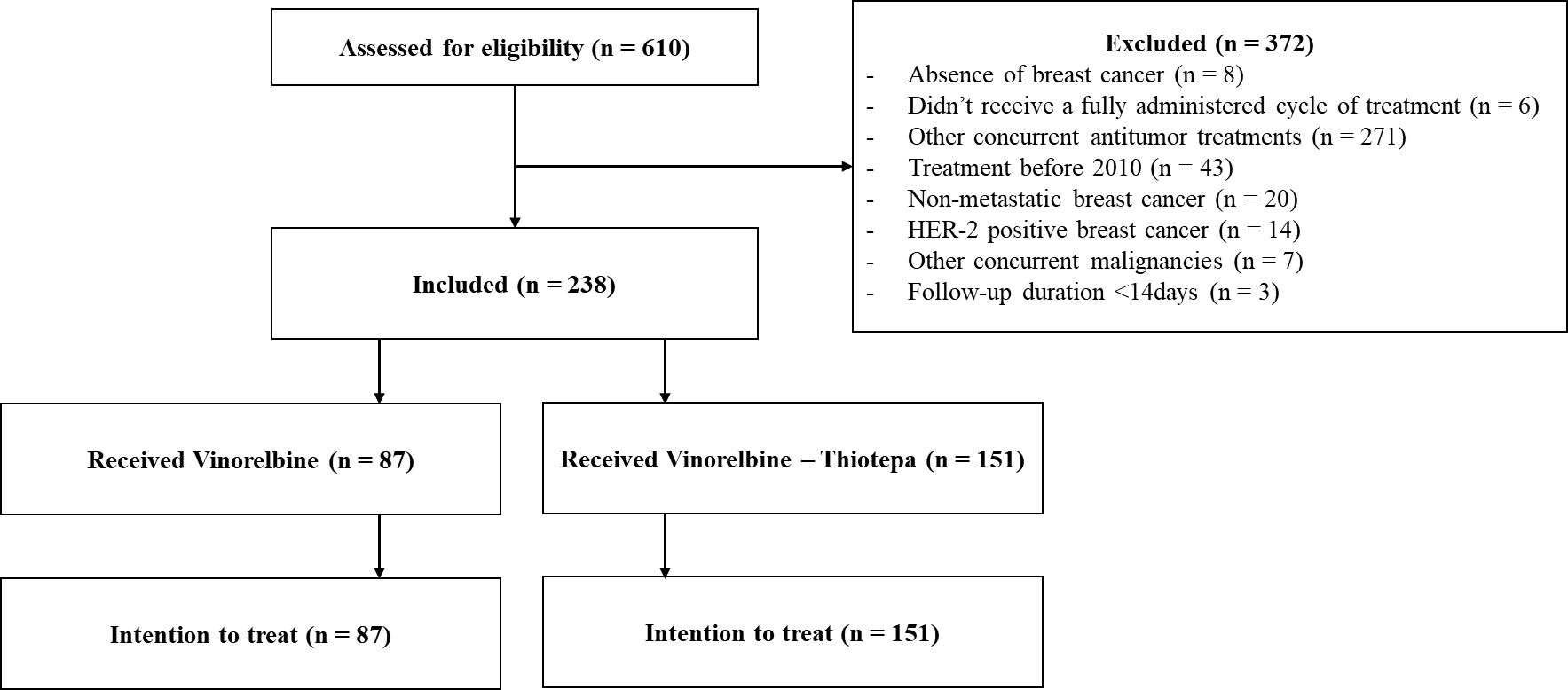


HER2: Human epidermal growth factor receptor 2

# **Supplementary Figure 3: propensity score distribution by treatment group.**


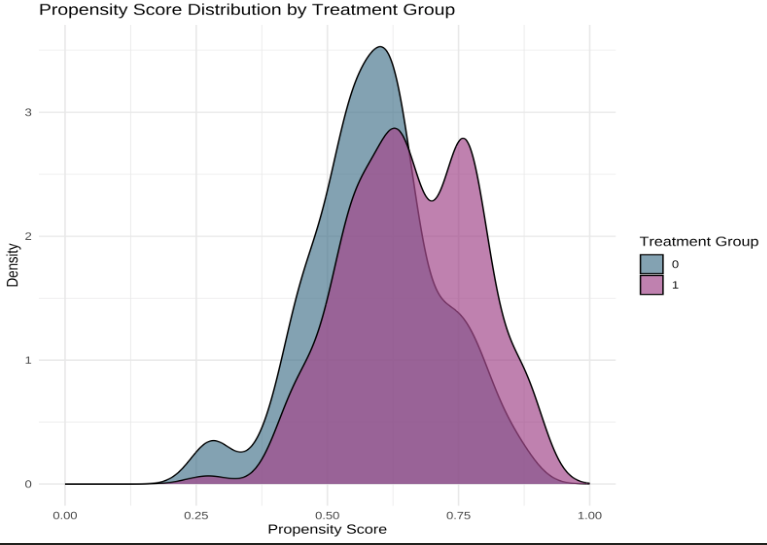


Visual assessment of the density of propensity score balance for each treatment group.

Treatment group = 0 for vinorelbine, 1 for vinorelbine + thiotepa

# **Supplementary Figure 4: quality check for inverse probability of treatment weighting**


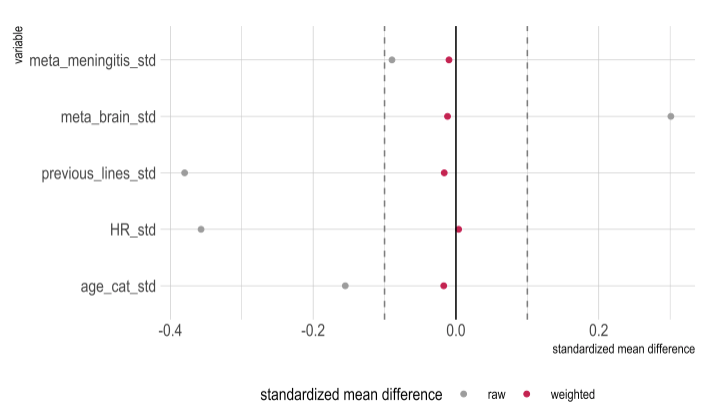


**-0.1**

**0.1**

Standardized mean difference for each variable before (raw, in grey) and after weighting (weighted, burgundy).

Abbreviations: cat: category ; HR: hormone receptor ; meta: metastasis ; std: standardized

# **Supplementary Figure 5: progression-free survival in the IPTW-adjusted hormone receptor-positive and the triple negative populations:**


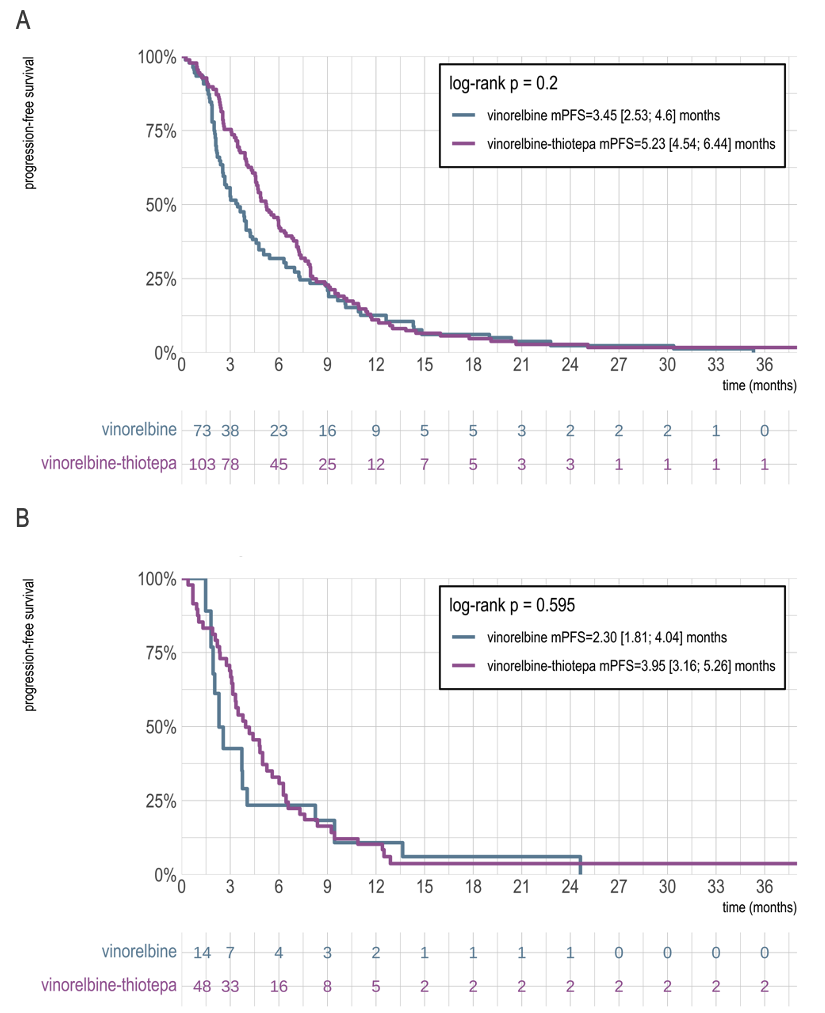


Kaplan-Meier Progression-Free Survival (PFS) curves for the (**A**) IPTW-adjusted HR+ population and the (**B**) TNMBC population.

mPFS: Median Progression-Free Survival; HR+: hormone receptor-positive; TNMBC: triple negative metastatic breast cancer; IPTW: inverse probability of treatment weighting.

# **Supplementary Figure 6: central nervous system progression-free survival (CNS-PFS) in the IPTW-adjusted CNS-metastasis population:**


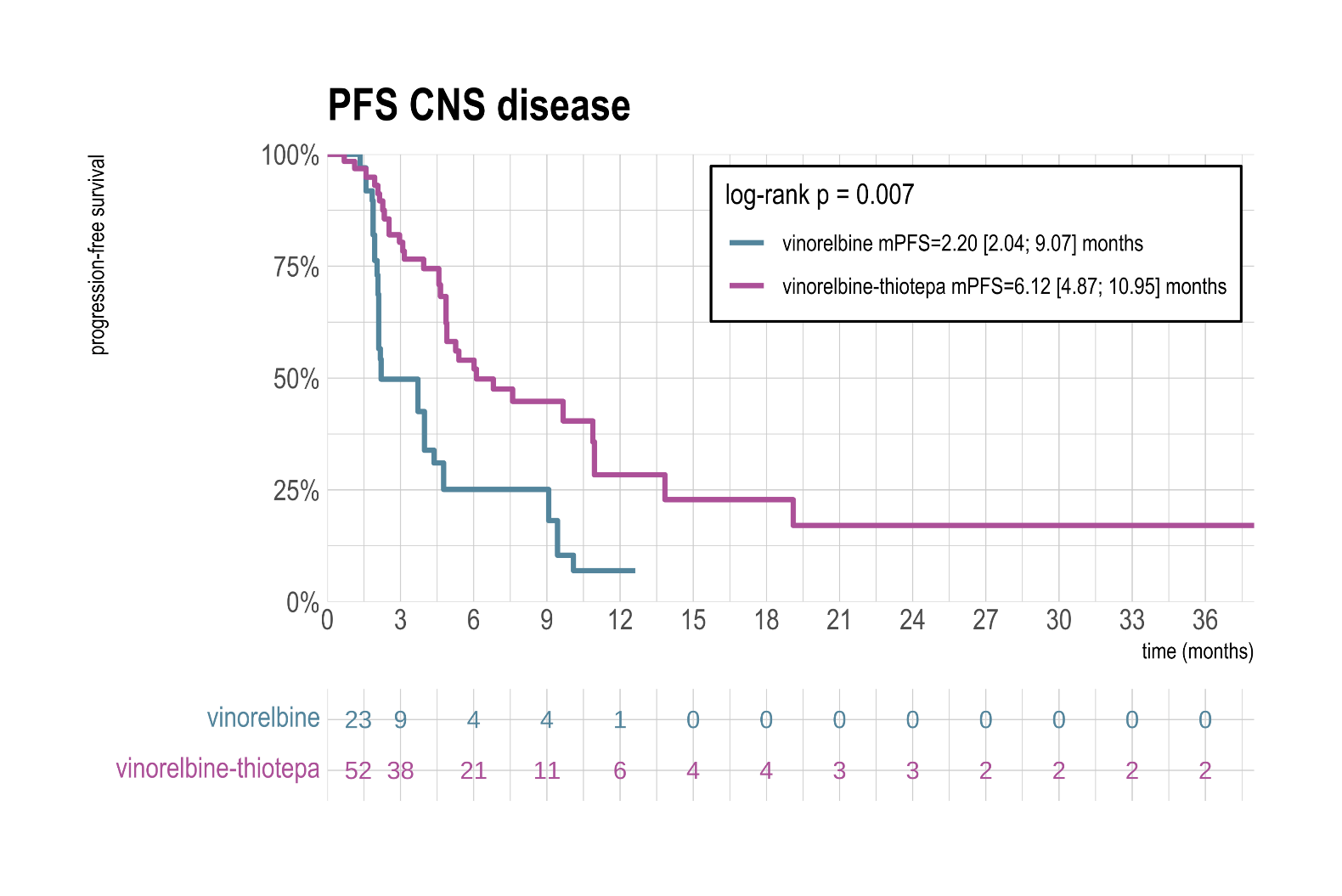


Kaplan-Meier Central Nervous System (CNS) Progression-Free Survival (PFS) curves for the CNS-metastasis population.

CNS: Central Nervous System; mPFS: Median Progression-Free Survival; IPTW: inverse probability of treatment weighting.
